# Visuospatial cognitive deficits in a mixed ALS/FTLD‐TDP Types A+B: A Case Report

**DOI:** 10.1002/alz70855_106144

**Published:** 2025-12-24

**Authors:** Haritha Vardhini Katragadda, Ali Ghaseminejad‐Bandpey, Mallory Keating, Mohammad Alhneif, Mariam Mojtabai, Mohamad Habes, Sudha Seshadri, Margaret E Flanagan, Kevin F. Bieniek

**Affiliations:** ^1^ Glenn Biggs Institute for Alzheimer's & Neurodegenerative Diseases, University of Texas Health Sciences Center at San Antonio, San Antonio, TX, USA; ^2^ Glenn Biggs Institute for Alzheimer's & Neurodegenerative Diseases, UT Health San Antonio, San Antonio, TX, USA; ^3^ Glenn Biggs Institute for Alzheimer's & Neurodegenerative Diseases, University of Texas Health Science Center, San Antonio, TX, USA

## Abstract

**Background:**

Amyotrophic lateral sclerosis (ALS) with frontotemporal dementia (FTD) is a neurodegenerative disorder characterized by an admixture of motor and cognitive/behavioral impairments (either concurrent or sequential). The predominant pathological hallmark is TDP‐43 proteinopathy, but it encompasses multiple frontotemporal lobar degeneration (FTLD) TDP‐43 subtypes, complicating both diagnosis and classification. The case report herein details a 69‐year‐old female with a history of cognitive decline, dysarthria, and behavioral changes, ultimately diagnosed with ALS‐FTD.

**Method:**

A 69‐year‐old right‐handed Caucasian female presented with decreased short‐term memory. Initial neuropsychological testing in 2019 revealed superior performance on the Dementia Rating Scale (DRS; 143/144) and Mini‐Mental State Exam (MMSE; 30/30), with mild impairment in visuospatial construction, visual‐motor organization, and visual memory, consistent with mild cognitive impairment (MCI). MRI revealed mild ischemic changes, more pronounced in the pons and left basal ganglia. No significant frontal or temporal lobar atrophy was observed, consistent with findings from 2017 MRI. Follow‐up testing in 2020 revealed mild to moderate impairments across multiple visuospatial cognitive domains, which were consistent with MCI. By late 2021, the patient developed progressive dysarthria, executive dysfunction, and behavioral changes (inappropriate), and pseudobulbar affect resulting in an antemortem diagnosis of ALS with FTD. While delusions are typically present in most cases, they were notably absent in this case.

**Results:**

At the age of 72, Postmortem neuropathology revealed changes consistent with mild cerebrovascular disease and Intermediate‐level Alzheimer's disease neuropathological changes (ADNC). Immunohistochemical analysis for phosphorylated TDP‐43 demonstrated skein‐like inclusions in the spinal cord as well as neocortical glial cytoplasmic inclusions, neuronal cytoplasmic inclusions, and dystrophic neurites, consistent with mixed FTLD‐TDP and ALS as the primary pathological findings. TDP‐43 pathology was not specific to a single FTLD‐TDP subtype, rather features of both Type A and Type B were present. Additionally, p62‐immunohistochemistry indicates possible C9ORF72 repeat expansion (with genetic testing pending).

**Conclusion:**

Mixed Type A+B cases are typically associated with C9ORF72 repeat expansions and prominent delusions. In contrast, the case presented here lacked delusions, with early disease features predominantly characterized by visuospatial deficits. The clinical presentation, along with overlapping pathological features of FTLD‐TDP subtypes A and B, underscores the atypical nature of this case relative to previously reported series.

